# *Meloidogyne* spp. in Eucalypts - Reproduction and Damage to Seedling Growth

**DOI:** 10.2478/jofnem-2023-0059

**Published:** 2024-01-23

**Authors:** José Olívio Lopes Vieira, Renata Cunha Pereira, Mariana Zandomênico Mangeiro, Ricardo Moreira Souza

**Affiliations:** Department of Entomology and Plant Pathology, Universidade Estadual do Norte Fluminense Darcy Ribeiro. Av. Alberto Lamego, 2000, Campos dos Goytacazes, Brazil, 28013-600

**Keywords:** *Corymbia citriodora*, *Eucalyptus* spp., host-parasitic relationship, nematode damage, resistance, root-knot nematode

## Abstract

Eucalypts are cultivated worldwide, but little is known about their status as hosts of root-knot nematodes (RKN) (*Meloidogyne* spp.). Moreover, information is scarce regarding the nature of the damage caused by RKN to eucalypt seedlings and trees. To investigate these aspects, we separately inoculated *Meloidogyne enterolobii*, *M. javanica* and *M. incognita* in seedlings of the world’s most cultivated eucalypts: *Eucalyptus dunni*, *E. grandis*, *E. cloeziana*, *E. camaldulensis*, *E. saligna*, *Corymbia citriodora*, and the hybrid *E. grandis* × *E. urophylla*. After six months of greenhouse cultivation, we assessed nematode reproduction and variables that expressed the seedlings’ shoot and root growth. We observed a diverse pattern of host statuses to RKN among the eucalypts, and all three *Meloidogyne* species reduced (*p* < 0.05) the root system mass, volume and length of *E. grandis*, *E. saligna* and the hybrid *E. grandis* × *E. urophylla*. Our results reaffirm previous reports indicating that RKN can delay the growth of seedlings in nurseries, who should thus adopt appropriate sanitary measures to avoid RKN establishment and spread. Moreover, the damage caused by RKN to eucalypts after just six months of cultivation suggests that the growth of eucalypt trees may be affected over the course of several years of cultivation.

Eucalypts are the most widely cultivated hardwood tree, found in over 100 countries and accounting for about 22 million hectares (Hua et al., 2002). Eucalypts are often cultivated in areas no longer suitable for other crops, in which the soil has low fertility and low levels of organic matter and microorganisms (Zhang et al., 2012). In such areas, eucalypt seedlings can suffer greater damage from plant pathogens (Naidoo et al., 2014). Fungi and bacteria are the main pathogens of eucalypts (Paine et al., 2011), but nematodes are also an emerging concern as parasites of eucalypt trees (Cruz et al., 2003) and seedlings (Khan, 2012; Bellé et al., 2018; Silva et al., 2020; Souza and Inomoto, 2021).

Globally, root-knot nematodes (RKN) (*Meloidogyne* spp.) are the most damaging nematode group (Perry et al., 2009). In susceptible plants, RKN can induce root galling, cause hormonal and nutritional imbalances, and cause yield losses. Even resistant plants can suffer damage when numerous second-stage juveniles (J_2_) invade and migrate through their root tissues. *Meloidogyne incognita* (Kofoid and White) Chitwood and *M. javanica* (Treub) Chitwood occur worldwide, while *M. enterolobii* (Yang and Eisenback) Rammah and Hirschmann is an emerging concern in several countries (Collett et al., 2021). These RKN are known to interact with soilborne fungi to form disease complexes. For instance, in guava (*P. guajava* L) a myrtaceae like eucalypts, *M. enterolobii* interacts with *Neocosmospora falciformis* (Carrión) L. Lombard and Crous, leading to guava decline (Gomes et al., 2011; Veloso et al., 2021).

*Eucalyptus dunni* Maiden, *E. grandis* Hill ex Maiden, *C. citriodora* Hook and the hybrid *E. grandis* × *E. urophylla* ST Blake have all been reported as resistant to *M. javanica* and *M. incognita* (Almeida et al., 2012; Silva et al., 2020; Souza and Inomoto, 2021). Nonetheless, the genetic variability of RKN suggests that more eucalypts should be assessed for their resistance/susceptibility to a larger range of *Meloidogyne* species and populations. Moreover, reports of the effects of RKN on the growth of eucalypt seedlings are scarce (Ferraz and Lordello, 1982; Silva et al., 2020). The growth of RKN-susceptible or RKN-resistant seedlings can be delayed if they are planted in areas previously cultivated with crops that harbored high RKN populations.

Hence, this study aimed i) to assess the host status of the world's most cultivated eucalypts to *M. enterolobii*, *M. javanica*, and *M. incognita*; and ii) to assess the impact of these *Meloidogyne* species on seedling growth. We hypothesized that a distinct host status (immune, resistant, or susceptible) would be found depending on the eucalypt and the nematode species involved, and that RKN would delay the seedling growth regardless of this host status.

To test these hypotheses, seedlings of six eucalypt species and one hybrid were inoculated with *M. enterolobii*, *M. javanica* or *M. incognita*, and then grown in a greenhouse over a period of six months. Nematode reproduction and variables related to the seedlings’ vegetative growth were assessed, and the data were analyzed.

## Materials and Methods

### Eucalypt cultivation

Eucalypt seeds were purchased from Caiçara Sementes (Brejo Alegre, Brazil). The substrate for seeding and seedling growth was prepared by mixing washed riverbed sand and soil (1:1). The substrate was disinfested in an Embrapa-type solar collector (Ghini, 2004) for three days under full sun before use. The temperatures that are reached inside the metal containers (60 to 80 °C) are known to eradicate both fungi and nematodes (Martins et al., 2003).

Seeding and seedling growth were conducted in plastic plant growth tubes (280 cm^3^) in a greenhouse (Supplementary Fig. 1). For each eucalypt species or hybrid, seven seeds were sown per tube, and thinned later to one seedling. The seedlings were irrigated once a day during winter, and twice in the summer. During the assay, the minimum and maximum daily temperatures were recorded locally. These temperatures averaged 17 and 30 ºC, respectively. No diseases or insect pests were observed during the assay.

### Experimental design, nematode sources, and inoculation

For each combination of eucalypt species or hybrid and *Meloidogyne* species, two treatments were assessed: nematode inoculation or no inoculation (control), with 10 replicate seedlings per treatment, for a total of 20 seedlings. The seedlings were arranged randomly in a tray of growth tubes.

*Meloidogyne javanica* and *M. incognita* were obtained from pure cultures maintained on tomato plants (*Solanum lycopersicum* L.) in a greenhouse at the Universidade Federal de Viçosa (Brazil). *Meloidogyne enterolobii* was obtained from a pure culture maintained on tomato and okra plants (*Abelmoschus esculentus* L.) in a greenhouse at the Universidade Estadual do Norte Fluminense Darcy Ribeiro (Brazil). The species’ identities were ascertained by morphology and esterase phenotyping (Esbenshade and Triantaphyllou, 1985).

To obtain the nematode inocula, infected tomato or okra roots were cleaned with tap water and submitted to nematode extraction using the method specified by Coolen and D’Herde (1972), but without adding kaolin to the blender. The resulting suspension was passed through 65- and 500-mesh sieves (with 250 and 25-μm openings, respectively). The nematode eggs and J_2_ retained on the 500-mesh sieve were suspended in water, and three 1-mL aliquots were counted on Peters’ slides under a stereomicroscope. For the three *Meloidogyne* species, the inoculum was calibrated to 50 eggs + J_2_/mL of water. The seedlings were inoculated 60 days after sowing, when two 5-cm-deep holes were made in the soil around each seedling, into which 10 mL of the inoculum (500 eggs + J_2_) was applied.

### Evaluation and data analysis

One hundred eighty days after inoculation, the seedlings were removed from the growth tubes and cut at the collar region. The shoot height (SH, in cm), stem diameter at the collar region (SD, in mm) and mass of the leaves after drying at 65 °C for 48 h (ML, in g) were all assessed. The root system was gently washed in tap water, and the following assessments were performed: length (RSL, in cm), measured from the collar until the most distal rootlet; fresh mass (RSM, in g); and volume (RSV, in cm^3^), measured by inserting the root system in a 50-mL graduated glass tube filled partially with water and measuring the displacement of water.

Nematodes were extracted from the roots and counted as described before. Eggs + J_2_ constituted the final nematode population (Pf). The nematode reproduction factor (RF) was determined by the Pf/500 ratio. For each combination of eucalypt species or hybrid and *Meloidogyne* species, the host status was determined according to Oostenbrink (1966), where RF = 0 denotes immunity; RF = 0.1 to 0.99 denotes resistance; and RF > 1 denotes susceptibility.

Data were submitted to the Shapiro-Wilk test to verify the normality of variances. Since the data were not normally distributed (*p* > 0.05), the treatments’ means were compared using the Mann-Whitney test at 5% significance (Mann and Whitney, 1947). The statistical analyses and graphs were generated using SigmaPlot 12.0 (Systat Software Inc., USA).

## Results

**Figure 1:**
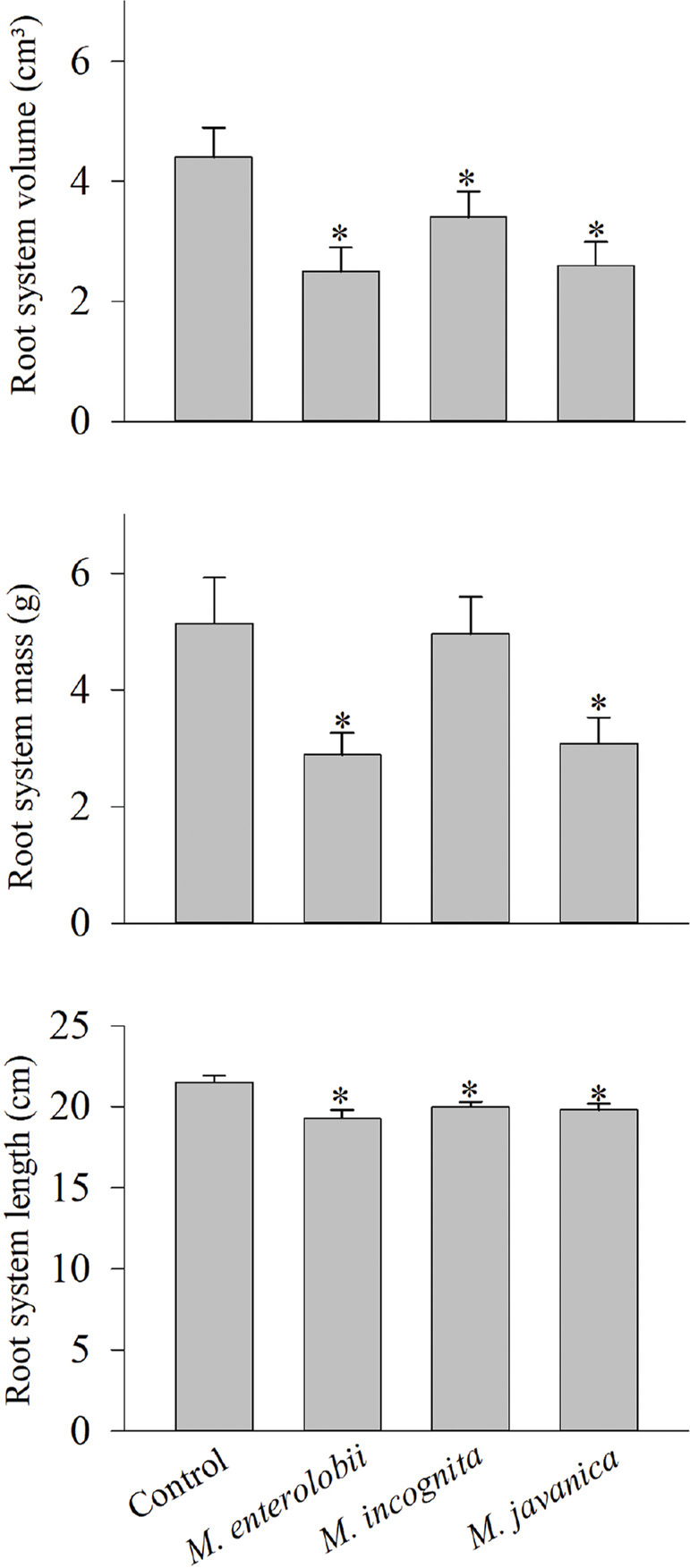
Effect of *Meloidogyne enterolobii*, *M. incognita*, and *M. javanica* on root system volume, mass, and length of *Eucalyptus grandis* seedlings grown in a greenhouse for six months. Values are the mean of 10 seedlings per nematode species. “*” denotes a significant difference relative to the control according to the Mann–Whitney test at 5% significance.

**Figure 2:**
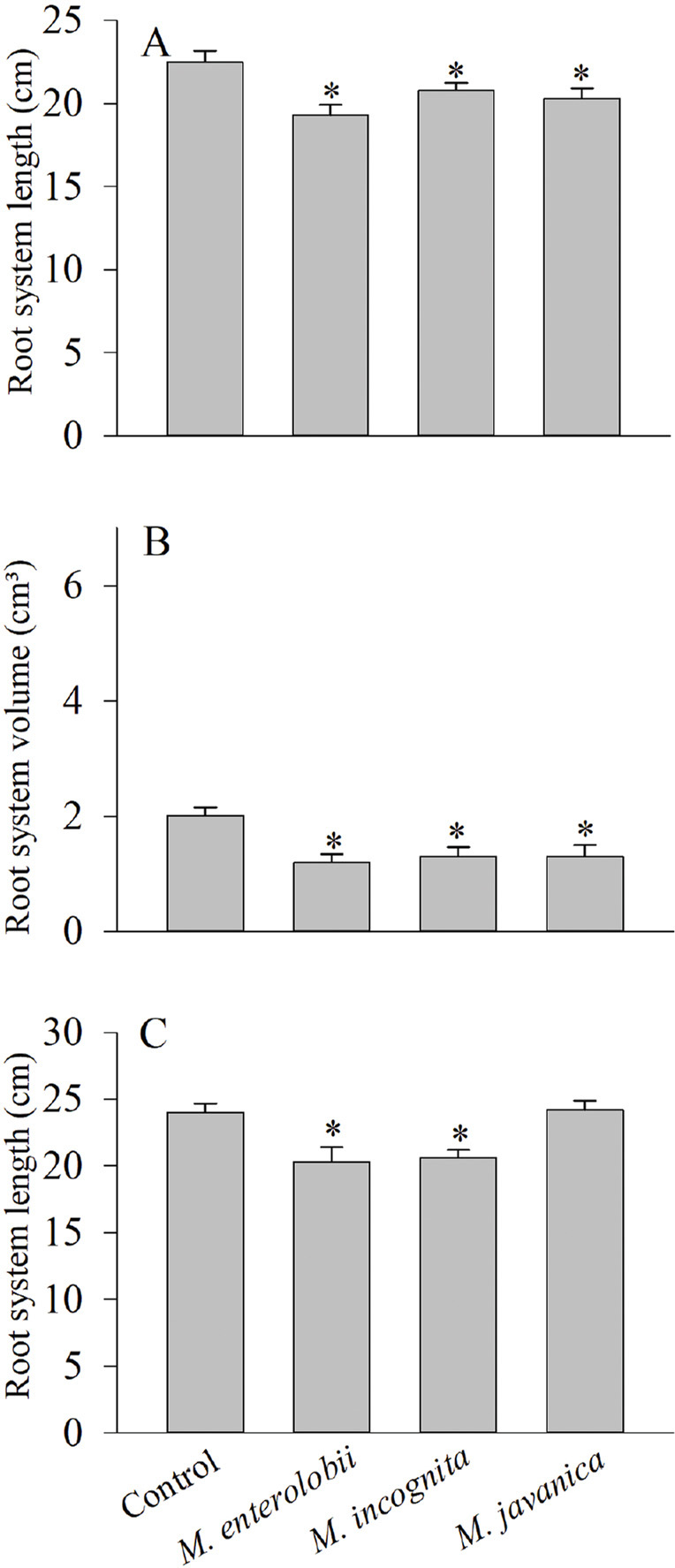
Effect of *Meloidogyne enterolobii*, *M. incognita*, and *M. javanica* on root system length and volume of eucalypt seedlings grown in a greenhouse for six months. A) *Eucalyptus saligna*. B, C) hybrid *E. grandis* × *E. urophylla*. Values are the means of 10 seedlings per eucalypt species or hybrid, per nematode species. “*” denotes a significant difference relative to the control according to the Mann-Whitney test at 5% significance.

*Corymbia citriodora*, *E. cloeziana* F. Muell., and *E. dunni* were resistant to *M. enterolobii*, *M. incognita*, and *M. javanica*, as the RF in these combinations was below 1 (Table 1). Moreover, none of the variables expressing seedling growth were affected by the nematodes (Supplementary Table 1). *Eucalyptus grandis* was resistant to all three *Meloidogyne* species, but the species did all have an effect (*p* < 0.05) on the growth of the seedlings’ root system (Fig. 1). *Eucalyptus camaldulensis* Dehnh was susceptible to *M. javanica* only (RF = 1.04), and no damage (*p* < 0.05) occurred to seedling growth. *Eucalyptus saligna* Smith was susceptible to *M. incognita* and *M. javanica*, but all three *Meloidogyne* species reduced RSL (*p* < 0.05) (Fig. 2A). The hybrid *E. grandis* × *E. urophylla* was susceptible to *M. enterolobii* and *M. javanica*, but all three RKN reduced the seedlings’ RSV and RSL (*p* < 0.05) (Figs. 2B,C).

**Table 1:** Reproduction factor and host status of *Eucalyptus* spp. and *Corymbia citriodora* to *Meloidogyne enterolobii*, *M. incognita*, and *M. javanica*, after cultivation in greenhouse for six months

**Species**	***M. enterolobii***	***M. incognita***	***M. javanica***
*E. camaldulensis*	0.22^a^ (R)^b^	0.52 (R)	1.04 (S)
*E. cloeziana*	0.60 (R)	0.46 (R)	0.62 (R)
*E. dunni*	0.50 (R)	0.82 (R)	0.74 (R)
*E. grandis*	0.38 (R)	0.62 (R)	0.42 (R)
*E. saligna*	0.94 (R)	4.82 (S)	3.44 (S)
*E. grandis × E. urophylla*	1.72 (S)	0.48 (R)	2.46 (S)
*C. citriodora*	0.94 (R)	0.90 (R)	0.54 (R)

a Values are the mean of 10 seedlings per eucalypt species or hybrid, per nematode species.

b R denotes resistance, and S denotes susceptibility according to the criteria of Oostenbrink (1966).

## Discussion

This study revealed a diverse pattern of host statuses and pathogenicities across different RKN-eucalypt interactions. With regard to host status, resistance was predominant, with the exception of *E. saligna* to *M. incognita* and *M. javanica*; *E. camaldulensis* to *M. javanica*; and the hybrid *E. grandis* × *E. urophylla* to *M. enterolobii* and *M. javanica*. This is the first report of susceptibility to RKN in *E. camaldulensis*, the hybrid *E. grandis* × *E. urophylla*, and *E. saligna*. In a previous report of RKN resistance in *E. saligna*, the relatively short period (90 days) chosen for nematode reproduction may have contributed to a RF < 1 (Ferraz and Lordello, 1982).

Regarding the pathogenicity of RKN, this is the first report of damage by *M. incognita* and *M. enterolobii* to *E. grandis*, and by *M. enterolobii*, *M. incognita*, and *M. javanica* to *E. saligna* and the hybrid *E. grandis* × *E. urophylla*. *Meloidogyne incognita* has previously been found to be pathogenic to *C. citriodora*, and *M. arenaria*, *M. javanica*, and *M. morocciensis* to *C. citriodora* and *E. grandis* (Ferraz and Lordello, 1982; Silva et al., 2020). Collectively, this indicates that RKN may be pathogenic to eucalypt seedlings regardless of the host status.

Taken together, our results on host status and pathogenicity suggest that in certain RKN-eucalypt interactions there may be tolerance (the ability to withstand growth despite susceptibility) or intolerance (sustaining damage despite being resistant). Nonetheless, it seems premature to assign such terms to RKN-eucalypt interactions. For a crop that may stand in the field for a dozen years, it seems advisable to carry out an additional field assay (1 to 2 years) to clarify this issue.

Presumably, the damage we observed to the seedlings’ root growth have consequences in the field. Indeed, the eucalypt root system, which can reach up to 60 meters deep, is crucial to sustaining the tree’s rapid growth (Whitehead and Beadle, 2004). It is plausible to think that planting eucalypt seedlings infected with RKN, or cultivating eucalypts in RKN-infested fields, may delay their growth, and years later reduce their productivity of wood, charcoal or other products.

In conclusion, our results highlight the need for more studies on the pathogenicity of RKN to eucalypts, particularly several months after the seedlings are transplanted to the field. Greater attention should be paid to the phytosanitary management of eucalypt nurseries, and the sampling of areas intended for eucalypt cultivation, in order to detect and quantify RKN.

## Supplementary Material

**Supplementary Table 1:** Mean ± error of shoot height (SH), stem diameter (SD), dry mass of leaves (ML), root-system mass (RSM), root system volume (RSV), and root-system length (RSL) of seedlings of *Eucalyptus* spp. and *Corymbia citriodora* when inoculated with *Meloidogyne enterolobii*, *M. incognita* or *M. javanica*, and cultivated in a greenhouse for six months.

**Treatments**	**SH (cm)**	**SD (mm)**	**ML (g)**	**RSM (g)**	**RSV (cm^3^)**	**RSL (cm)**
*Eucalyptus camaldulensis*						
*Meloidogyne enterolobii*	37.40 ± 0.72^*a*^	3.38 ± 0.10	0.76 ± 0.10	3.06 ± 0.44	4.0 ± 0.25	21.4 ± 0.78
*Meloidogyne incognita*	37.20 ± 0.79	3.62 ± 0.12	0.89 ± 0.11	2.99 ± 0.24	2.9 ± 0.37	21.2 ± 0.84
*Meloidogyne javanica*	34.50 ± 0.57	3.52 ± 0.11	0.89 ± 0.11	3.32 ± 0.31	2.8 ± 0.55	20.20 ± 0.42
Control	39.01 ± 0.88	3.39 ± 0.10	0.92 ± 0.14	3.06 ± 0.23	4.2 ± 0.41	21.4 ± 0.55
*Corymbia citriodora*						
*Meloidogyne enterolobii*	33.30 ± 0.53	3.01 ± 0.07	0.81 ± 0.07	4.23 ± 0.15	4.0 ± 0.33	20.0 ± 0.59
*Meloidogyne incognita*	34.06 ± 0.42	3.03 ± 0.09	0.70 ± 0.11	2.53 ± 0.17	3.8 ± 0.35	20.5 ± 0.22
*Meloidogyne javanica*	30.60 ± 0.69	2.90 ± 0.08	0.69 ± 0.06	4.13 ± 0.19	3.1 ± 0.31	20.0 ± 0.47
Control	33.33 ± 0.53	3.26 ± 0.08	0.81 ± 0.07	4.15 ± 0.14	4.5 ± 0.34	20.9 ± 0.48
*Eucalyptus cloeziana*						
*Meloidogyne enterolobii*	28.61 ± 0.93	3.26 ± 0.08	1.00 ± 0.14	2.98 ± 0.12	3.6 ± 0.55	18.8 ± 0.28
*Meloidogyne incognita*	27.90 ± 0.97	3.30 ± 0.11	1.10 ± 0.26	3.26 ± 0.22	3.4 ± 0.39	19.2 ± 0.38
*Meloidogyne javanica*	28.70 ± 0.97	3.25 ± 0.12	1.07 ± 0.14	4.98 ± 0.13	4.9 ± 0.50	18.8 ± 0.32
Control	32.50 ± 1.00	3.48 ± 0.09	1.26 ± 0.12	4.38 ± 0.14	3.8 ± 0.47	19.2 ± 0.24
*Eucalyptus dunni*						
*Meloidogyne enterolobii*	38.51 ± 0.76	4.18 ± 0.14	1.2 ± 0.16	2.72 ± 0.42	3.6 ± 0.38	18.8 ± 0.23
*Meloidogyne incognita*	37.17 ± 0.84	4.24 ± 0.12	0.96 ± 0.15	3.45 ± 0.47	3.2 ± 0.39	20.3 ± 0.38
*Meloidogyne javanica*	41.00 ± 0.81	4.27 ± 0.13	1.0 ± 0.11	3.07 ± 0.49	3.0 ± 0.51	18.8 ± 0.33
Control	41.20 ± 0.70	4.12 ± 0.12	1.23 ± 0.11	4.99 ± 0.44	4.6 ± 0.39	19.7 ± 0.39
*Eucalyptus grandis*						
*Meloidogyne enterolobii*	34.28 ± 0.78	3.44 ± 0.13	0.48 ± 0.07	2.89 ± 0.37*	2.50 ± 0.40*	19.3 ± 0.49*
*Meloidogyne incognita*	36.78 ± 0.69	3.81 ± 0.10	0.83 ± 0.09	4.96 ± 0.64	3.40 ± 0.43^*^	20.0 ± 0.29^*^
*Meloidogyne javanica*	36.56 ± 0.78	3.97 ± 0.11	0.45 ± 0.05	3.08 ± 0.45^*^	2.60 ± 0.39^*^	19.8 ± 0.39^*^
Control	36.52 ± 0.92	3.84 ± 0.12	0.60 ± 0.07	5.14 ± 0.78	4.40 ± 0.49	21.5 ± 0.41
*Eucalyptus saligna*						
*Meloidogyne enterolobii*	24.13 ± 0.62	3.75 ± 0.11	1.3 ± 0.16	4.86 ± 0.94	2.3 ± 0.61	19.0 ± 0.63^*^
*Meloidogyne incognita*	24.18 ± 0.65	3.82 ± 0.11	1.37 ± 0.13	7.87 ± 1.23	3.9 ± 0.76	20.8 ± 0.45^*^
*Meloidogyne javanica*	24.85 ± 0.59	3.49 ± 0.10	1.05 ± 0.14	5.73 ± 1.13	2.4 ± 0.57	20.3 ± 0.61^*^
Control	27.52 ± 0.54	3.58 ± 0.53	1.25 ± 0.15	6.74 ± 1.08	3.1 ± 0.49	22.5 ± 0.67
*Eucalyptus grandis* × *Eucalyptus urophylla*						
*Meloidogyne enterolobii*	20.30 ± 0.89	3.60 ± 0.10	1.06 ± 0.09	3.43 ± 0.5	1.20 ± 0.13^*^	20.3 ± 1.01^*^
*Meloidogyne incognita*	20.60 ± 0.59	3.75 ± 0.10	1.07 ± 0.17	2.41 ± 0.44	1.30 ± 0.15^*^	20.6 ± 0.63^*^
*Meloidogyne javanica*	24.26 ± 0.58	4.19 ± 0.27	1.32 ± 0.15	3.16 ± 0.46	1.30 ± 0.19^*^	24.2 ± 0.53
Control	27.67 ± 0.84	3.54 ± 0.42	1.19 ± 0.16	3.74 ± 0.39	2.01 ± 0.20	24.0 ± 0.66

a Values are the mean of 10 seedlings per eucalypt species or hybrid, per nematode species.

“*” Denotes a significant difference in relation to the control according to the Mann–Whitney test at 5% significance.
